# Sub-threshold micropulse laser treatment reduces inflammatory biomarkers in aqueous humour of diabetic patients with macular edema

**DOI:** 10.1038/s41598-019-46515-y

**Published:** 2019-07-11

**Authors:** Edoardo Midena, Alessandra Micera, Luisa Frizziero, Elisabetta Pilotto, Graziana Esposito, Silvia Bini

**Affiliations:** 1 Department of Ophthalmology, University of Padova, Padova, Italy; 2 IRCCS – Fondazione Bietti, Rome, Italy

**Keywords:** Proteomic analysis, Eye diseases, Diabetes complications

## Abstract

Subthreshold micropulse laser (SMPL) is a tissue-sparing technique whose efficacy is demonstrated for diabetic macular edema (DME) treatment. However, its mechanism of action is poorly known. A prospective observational study was performed on naïve DME patients treated with SMPL, to evaluate the changes of aqueous humor (AH) inflammatory and vaso-active biomarkers after treatments. AH samples of eighteen DME eyes were collected before and after SMPL. Ten non-diabetic AH samples served as controls. Full ophthalmic evaluation, spectral domain optical coherence tomography (SD-OCT) and fluorescein angiography were performed in DME group. Glass chip protein array was used to quantify 58 inflammatory molecules. Central retinal thickness (CRT) and visual acuity were also monitored. Several molecules showed different concentrations in DME eyes versus controls (p value < 0.05). *Fas Ligand (FasL)*, *Macrophage Inflammatory Proteins (MIP)*-*1α*, *Regulated on Activation Normal T Cell Expressed and Secreted (RANTES) and Vascular Endothelial Growth Factor (VEGF)* were increased in DME at baseline versus controls and decreased after SMPL treatments (p < 0.05). CRT reduction and visual acuity improvement were also found. Inflammatory cytokines, mainly produced by the retinal microglia, were significantly reduced after treatments, suggesting that SMPL may act by de-activating microglial cells, and reducing local inflammatory diabetes-related response.

## Introduction

### Pathophysiology of diabetic macular edema

Diabetic macular edema (DME) is a common complication of diabetic retinopathy (DR), representing the main cause of visual impairment in these patients^1–3^. It can occur at any stage of DR and, although widely investigated, its pathogenesis is still controversial^4^. Several mechanisms have been hypothesized: mainly the breakdown of the blood-retinal barriers^4^. However, DR and DME cannot be considered anymore as pure microvascular complications of diabetes mellitus (DM). The role of inflammation has recently received great attention as an upstream factor in the pathophysiology of DR and DME^5,6^. A “low grade” chronic inflammation inside the retina has been hypothesized, and demonstrated *in vivo*^4–7^, as a driving factor for the development of both DME and proliferative DR.

### Role of retinal glial cells

Retinal glial cells (GLC) are the main actors of retinal inflammatory processes^8,9^. They are mainly represented by Müller cells and astrocytes, called macroglia, and by the microglia^8,9^. The role of GLC has been considered mainly structural in the past, whereas an increasing body of scientific evidence shows that they actively maintain the homeostasis of the retinal environment. The main role of Müller cells, biologically connecting retinal neurons and vessels, is to maintain retinal water control. They also participate in the inflammatory response, especially when activated by diabetes^8^. Microglial cells (MGC) are considered the local immune cells of the retina^8^, similarly to the central nervous system (CNS) microglial cells. They are activated by stress conditions, such as DM, and are able to change their morphology and function. In the healthy retina, MGC are predominantly localized in the inner retinal layers, in a ramified resting status^9^. When activated for example by diabetes, MGC turn into an ameboid form and gain motility. MGC than migrate toward, from the inner to outer retina, and release pro-inflammatory and vaso-active substances, such as vascular endothelial growth factor (VEGF), contributing to the local inflammatory response followed by increasing vascular permeability^9,10^.

### Laser therapy for DME

Because inflammation is a leading cause of DM, therapeutic strategies are aimed to control the over expression of inflammatory cytokines^10^. Laser photocoagulation has historically represented the main option for the treatment of DME^11,12^. Subthreshold Micropulse Laser (SMPL) represents a relatively new retinal laser technique^13–17^. Unlike conventional laser photocoagulation, SMPL is a tissue-sparing technique: it avoids protein coagulation (therefore it is not a photo-coagulation) and prevents retinal scars, allowing retinal anatomic and functional preservation^13^. It has been hypothesized that SMPL, by inducing a controlled thermal elevation of the retinal tissue, is able to selectively stimulate the retinal pigment epithelium (RPE)^12,18^. Nevertheless, the precise metabolic changes induced by SMPL are poorly known. The proteomic approach on samples of vitreous body and aqueous humour (AH) of diabetic eyes has significantly contributed to elucidate the pathophysiology of DME and the effects induced by some different treatments^7,19–29^. Our group has already demonstrated that SMPL reduces AH biomarkers of Müller cells in treated DME eyes^29^. The aim of this study was therefore to evaluate if SMPL is also able to influence the concentration of the inflammatory AH biomarkers, specifically produced by MGC in DME eyes. Visual acuity and retinal thickness changes of DME patients, measured by spectral domain optical coherence tomography (SD-OCT), were also evaluated.

## Materials and Methods

### Population

A prospective interventional study was performed on DME eyes. The major inclusion criteria were: women or men with type 2 DM; recent HbA1c ≤10%; presence of previously untreated DME, central retinal thickness (CRT) ≤400 μm at Spectral Domain Optical Coherence Tomography (SD-OCT); best corrected visual acuity (BCVA) ≥35 score on ETDRS chart (logMAR 1.0, Snellen 20/200). The main exclusion criteria were: proliferative or severe non proliferative DR; any previous macular treatment (laser photocoagulation, intravitreal injections, vitreo-retinal surgery), any concomitant local treatment (steroid or non-steroidal drugs), post-surgical macular edema of any other etiology; refractive error ≥6 diopters; previous diagnosis of glaucoma or ocular hypertension; concomitant retinal diseases; major neurodegenerative disorders (multiple sclerosis, Alzheimer’s disease etc.); uncontrolled systemic blood pressure, renal failure or systemic disease potentially influencing the protein expression in body fluids; any intraocular surgery in the last 12 months before the beginning of the study; significant media opacity precluding fundus examination. All diabetic patients underwent a full ophthalmologic evaluation, including slit lamp biomicroscopy, intraocular tension measurement, ophthalmoscopic examination and SD-OCT at baseline (before treatment) and after 3, 6, 9 and 12 months during follow-up visits. Fluorescein angiography was also performed at baseline and at 12 months. Ten non-diabetic patients without any eye disease other than cataract were included as controls^30^. In both groups of examined patients (DME and non-diabetic control group), a certain degree of lens opacification (cataract), not precluding a correct fundus examination and imaging, was present. In non-diabetic control group, AH was sampled once, before standard cataract surgery. Exclusion criteria for non-diabetic control cases were strict: patients with any previous or present ocular disorder, any previous ocular surgical procedure, recent (6 months) local treatment of the eye, or patients affected by any significant systemic disease, were excluded from the study, in order to avoid confounding data. Therefore, also control eyes underwent a full ophthalmologic evaluation, in order to exclude any intraocular disorders other than cataract. The informed consent was obtained for each patient of both groups (DME and non-diabetic subjects), and the research was carried out in accordance with the Declaration of Helsinki regarding experimentation involving human tissue. The approval from the Ethics Committee for Clinical Practice (CESC) of the AOP (Azienda Ospedaliera di Padova) for the study was obtained, with protocol number 3194/AO/14.

### Spectral domain optical coherence tomography

SD-OCT was performed using Spectralis (Spectral HRA + OCT; Heidelberg Engineering, Heidelberg, Germany). Central retinal thickness (CRT) was measured on an En-Face macular map 20° × 20° (5.90 × 5.90 mm). Ninety-seven horizontal scans 60 μm apart were obtained. Automatic real time tracking was on for each acquisition at 50 frames. A linear scan at 180°, length 6 mm was also obtained. Mean retinal thickness was calculated for each of the 9 ETDRS areas (central circle 1 mm diameter, and two external rings 3 and 6 mm diameter). Mean retinal thickness of the entire macular map was measured at each visit. Follow-up modality was set in order to obtain a perfect comparison among the OCT maps and linear scans acquired during one-year follow-up.

### Treatment protocol

Pupillary dilation and topical anesthesia were performed before SMPL and a Mainster Focal/Grid (Ocular Instrument, Bellevue, WA) lens was used. SMPL treatment was performed with a 577-nm yellow light (Iridex IQ 577; Laser System Iridex Corp, CA), 5% duty cycle of 0.2 seconds, power 250 mW, as already published by our group^14,16^. No titration was performed, and the same parameters were applied to all patients by a single operator, in order to guarantee comparable results. SMPL was performed in high-density fashion, with multiple and fully confluent spots over the entire area of retinal thickening^31^. All DME-eyes underwent treatment just after baseline visit. Retreatment was applied 3 months apart, if central subfield OCT macular thickness was ≥300 μm and/or thickness reduction <50% from baseline in a subfield measured at OCT and/or reduction ≥5 letters on the ETDRS chart. A maximum of 4 treatments for each DME patient was applied during the study duration.

### Sample collection, storage and total protein analysis

The AH samples of DME eyes were collected at baseline, and at 1, 3 and 12 months post first SMPL application, while the sampling of the non-diabetic control group was performed only once, as no changes of proteins’ concentration were expected through the time. All patients underwent the standard preoperative procedure: peri-ocular skin disinfection with povidone-iodine 10% (ESO-JOD, ECOLAB, Agrate Brianza, Italy), instillation of sterile lidocaine 4% (Alpha Intes, Napoli, Italy), irrigation of conjunctival sac with povidone-iodine 5% (Oftasteril, Alpha Intes) for two minutes and washing of the eye with Balanced Salt Solution (BSS; Alcon, Fort Worth, TX, USA). From the anterior chamber, AH (150–200 μL) was aspirated with an insulin syringe (31-gauge needle). AH samples were then collected in a single vial containing 10 μL of a cocktail of protease inhibitors (Pierce Biotechnology, Rock-ford, IL, USA) and quickly stored at −70 °C until analysis. The total protein content was quantified with a digital spectrophotometer (NanoDrop; Thermo Fisher Scientific Inc., Waltham, MA, USA) and protein concentrations were calculated by means of the linearized standard curve (BSA) and the A280 software. Thereafter, AH samples underwent sonication (VibraCell; Sonics, Newton, CT, USA) and centrifugation to collect clear supernatant (13000 rpm/7 min).

### Inflammatory profile of protein array

Considering the still unknown mechanism of action of SMPL, the aim of this study was to analyze a wide range of molecules that would hypothetically be influenced in their concentration by this type of laser treatment. Therefore, a customized protein array on glass-chips, from RayBiotech^TM^ technology, established by the manufacturer (Norcross, GA, USA), was used. For the purpose of this study, ELISA was not used immediately, but planned for the further evaluation of the single proteins showing significant changes in this first step analysis with glass-chip array. The array-map included 58 markers. Normalized and pre-diluted AH samples were loaded on chip arrays, according to the manufacturer’s instruction, including target, positive, negative and internal control spots. Both diabetic and control groups were processed in parallel. After an overnight incubation at 4 °C, the array slides were washed and exposed to a biotinylated antibody mixture followed by a cy3-streptavidin labeling solution. All steps were performed under orbital shaking (Certomat II, Sartorius AG, Göttingen, Germany), with hybridization/washing solutions provided by the kit. Finally, glass-slides were washed once with MilliQ water, spin-dried and acquired with GenePix 4400 Microarray scanner (Molecular Devices LLC, Sunnyvale, Silicon Valley, CA, USA). To obtain appropriate Cy3/Cy5 (specific/background signals) images, the slides were scanned over previously validated acquisition parameters and procedures. The fluorescence signals were acquired with the GenePix 4100 microarray scanner (Molecular Devices LLC, Sunnyvale, CA, USA) equipped with the GENEPIX pro 3.0 software (Axon Instruments, Foster City, CA, USA). All comet tails were ignored and only median signal values obtained using the same setting were used for the identification of any biomarker variation. An inter- and intra–assay coefficient of variability limit of ≤10% was set for the study, and a 1.5-fold increase or ≤0.65-fold decrease in signal intensity was considered to guarantee specific signals above background. Fluorescent signals were analyzed and fold changes were generated (pathological/control ratio). The plot of proteins included several interleukins (IL-1β, 4, 6, 8, 10, 11, 12p40, 12p70, 13, 17, 21), Tumor Necrosis Factor (TNF)α, TNFβ, Interferon (INF)γ, Eotaxin, Eotaxin-2, Tissue Inhibitor of Metalloproteinase (TIMP) 1, TIMP-2, TIMP-3, TIMP-4, TNFα converting enzyme (TACE), Intracellular Adhesion Molecule (ICAM)-1, ICAM-2, ICAM-3, Vascular Cell Adhesion Molecule (VCAM)-1, Neural-Cell Adhesion Molecule (NCAM)-1, Osteopontin, Insulin, regulated and normal T cell expressed and secreted (RANTES), Macrophage Inflammatory Protein (MIP)-1α, MIP-1β, MIP-1δ, MIP-3α, MIP-3β, Toll Like Receptor (TLR)-2, Monocyte Chemo-attractant Protein (MCP)-1, IP-10, Glial cell Derived Neurotrophic Factor (GDNF), Brain-Derived Neurotrophic Factor (BDNF), NeuroTrophin (NT)-3, NT-4, Granulocyte-Colony Stimulating Factor (G-CSF), M-CSF, Placental Growth Factor (PlGF), Nerve Growth factor (NGF), VEGF, TGF β1, Platelet Derived Growth Factor (PDGF)-BB, basic Fibroblast Growth Factor (bFGF), Epidermal Growth Factor (EGF), soluble Tumor Necrosis Factor Receptor (sTNF R)-I, sTNFR-II, VEGF Receptor (VEGF-R)1, VEGF-R2, Insulin-like Growth Factor (IGF)-1, Fas Ligand (FAS L), β2-microglobulin (β2M), Albumin. In order to minimize intra- and inter-assay variability, a single tester handled all the material and followed all the phases of the experiment.

### Statistical analysis

Results were reported as mean value ± standard deviation. Statistically significant variations of CRT between baseline and follow-up visits, in DME group, were tested using Wilcoxon Signed Rank test. The comparison of AH proteins’ concentration in DME eyes at 1, 3 and 12 months and in non-diabetic control group was made, for each protein, by means of Wilcoxon-Mann-Whitney test. Changes in protein expression in diabetic samples with DME at 1, 3 and 12 months were separately compared to baseline samples and to non-diabetic control cases, and were tested by Wilcoxon signed rank sum test. For these two last analyses Benjamini-Hochberg procedure for multiple-testing correction has been applied choosing a False-Discovery-Rate (FDR). FDR inferior to 20% was set to accept results as statistically significant. All the analyses have been made using SAS® 9.3 statistical software (SAS-Institute, Cary NC, USA) on personal computer. P-value has been interpreted as statistically significant when <0.05 where not otherwise specified.

## Results

### Demographic characteristics

Eighteen diabetic patients (DM type 2), with non-proliferative DR and DME with CRT below 400 microns were enrolled in this study. Ten non-diabetic patients, planned to undergo cataract surgery, were also enrolled and served as non-diabetic controls. For each patient one eye was considered in the study analyses. All enrolled patients were selected as having the least number of concomitant systemic disorders, such as hypertension, renal impairment or failure, and dyslipidemia. The general characteristics of both study groups are listed in Table 1. No statistically significant differences were found between the two groups (Table 1).

### Functional and morphological outcomes

Mean BCVA of DME group at baseline was 77.4 ± 10.1 letters on ETDRS score (0.152 ± 0.021 logMar). All DME eyes were treated at baseline (after sampling AH) and re-treated at each time point (3, 6 and 9 months) of the study, as they satisfied the re-treatment criteria (see materials and methods). A variable number of laser spots depending on the extension of macular edema. BCVA showed a progressive improvement in number of letters at ETDRS charts compared to baseline, for all the study period: +2.9 ± 4 at 3 months, (+4.6 ± 8.3 at 6 months, (+5.3 ± 8.5 at 9 months and (+5.6 ± 9 at the end of study (12 months). At 3 and 9 months after tretaments a statistically significant increase was found (p = 0.047 for both). CRT showed a progressive reduction at each time point during study period. At baseline CRT was 370 ± 37.4 μm, the mean change at 3 months was −14.9 ± 48.9 μm, −12 ± 44.21 at 6 months, −23 ± 36.9 μm at 9 months and −25 ± 40.2 μm at 12 months. Despite the reduction of mean CRT value, the statistically significance was borderline in each time point compared to baseline (p = 0.062 at 9 months and p = 0.078 at 12 months). Comparing the results of fluorescein angiography at 12 months to baseline, no progression of DR or presence of new ischemic areas were found.

### Array analysis

**Figure 1:**
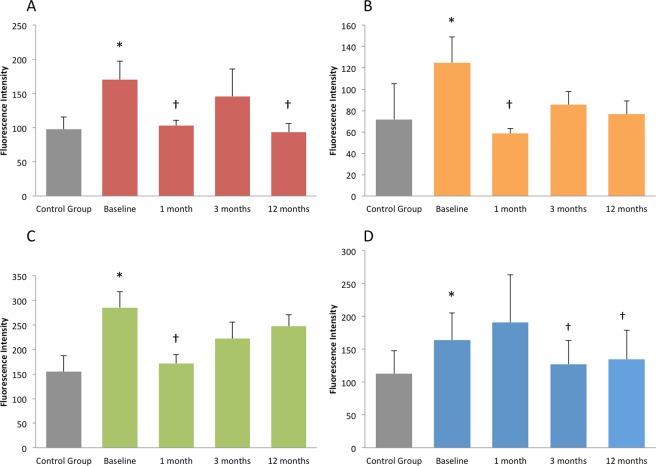
Representation of proteins’ concentration in non-diabetic subjects (control group) and in DME eyes at baseline and 1, 3 and 12 months after treatments with subthreshold micropulse laser. (**A**) Representation of RANTES expression; (**B**) representation of FasL expression; (**C**) representation of MIP1α expression; (**D**) representation of VEGF expression. * Indicates statistically significant difference compared to control group, † indicates statistically significant difference compared to baseline. *Abbreviations:* DME: diabetic macular edema; FasL: Fas Ligand; MIP1α: Macrophage Inflammatory Proteins; RANTES: Regulated on Activation Normal T Cell Expressed and Secreted; VEGF: vascular endothelial growth factor.

Total protein content in the AH samples remained stable during follow up. At baseline, seven proteins showed a significant change of concentration in AH samples of DME group compared to non-diabetic controls (see Tables 2 and 3). Among those, Osteopontin, TIMP 1 and TIMP2 were significantly reduced in DME eyes at baseline versus controls, a further reduction of TIMP2 at 12 months versus baseline was also found (see Table 2). Four proteins, RANTES, FasL, MIP1α and VEGF, showed an increased concentration in DME group at baseline versus non-diabetic controls (Table 3 and Fig. 1) and a significant reduction after treatments at 1 and/or 3 and/or 12 months (Table 4 and Fig. 1). Noteworthy, in DME group, most of the protein concentrations significantly reduced after SMPL treatments during the follow up visits versus baseline values (see Table 4). Even if mean concentration of these proteins at baseline in DME group was increased versus control group, a statistically significant difference was not fully detected. Correlations among each dosed molecule and its changes during the study were correlated with retinal thickness measured on OCT, not only CRT but also single retinal layers changes. However, despite some statistically significant data, these were inconstant trough time points and were therefore considered unreliable for further considerations.

**Table 1:** General characteristics of the study population and comparison between diabetic group and healthy controls.

	DME baseline (tot. 18)	Controls (tot. 10)	P-value*
Mean Age, mean ± SD	63 ± 8.7	69 ± 9.8	*0*.*515*
Sex, M/F	10/8	6/4	*0*.*819*
Presence of Hypertension, %	60%	50%	*0*.*569*
*BP Max*, *mean* ± *SD*	133.34 ± 10.33	128 ± 5.7	*0*.*559*
*BP Min*, *mean* ± *SD*	76.67 ± 7.52	82.6 ± 4.88	*0*.*386*
BMI, Kg/m^2^ mean ± SD	24.13 ± 1.89	26.56 ± 3.47	*0*.*350*
Cholesterolemia, mg/dl	188.2 ± 88.79	174.8 ± 38.66	*0*.*860*
Triglicerydes, mg/dl	133 ± 36.77	140.75 ± 34.33	*0*.*831*
DM duration, yrs, mean ± SD	15.2 ± 10.0	NA	NA
HbA1c, %, mean ± SD	7.4 ± 2.6%	NA	NA
Diabetes Treatment			
Insulin, n (%)	8 (44.45%)	NA	NA
Oral tablets, n (%)	9 (50%)	NA	NA
Diet, n (%)	1 (5.56%)	NA	NA

*Abbreviations:* DME: diabetic macular edema; M: males; F: females; BP: blood pressure; BMI: body mass index; DM: diabetes mellitus; NA: non applicable;

*Chi-square test p-value for sex and presence of hypertension; Wilcoxon-Mann-Whitney otherwise.

**Table 2:** Aqueous humour concentration of three proteins in healthy subjects (controls) and in eyes with DME at baseline and after treatment with SMPL.

Protein	Controls	DME Group							
		Baseline	P-value*	1 month	P-value§	3 months	P-value§	12 months	P-value§
Osteopontin	8907.2 (13143.2)	548.8 (588.5)	**0**.**007**	669.7 (1054.6)	—	686.3 (691.2)	—	400.0 (384.0)	—
TIMP1	473.9 (457.8)	141.5 (102.3)	**0**.**027**	161.6 (165.2)	—	150.9 (149.7)	—	89.3 (53.3)	—
TIMP2	28319.0 (28054.8)	474.1 (782.3)	**0**.**006**	598.1 (1391.3)	—	392.7 (750.1)	—	173.8 (286.6)	**0**.**013**

The results are reported as mean values and standard deviation in brackets of fluorescence intensities signals, measured on glass chip array. P-value = Wilcoxon-Mann-Whitney raw p-value; statistically significant results have been reported in bold characters. The concentration of each protein was significantly reduced in diabetic eyes at baseline versus controls.

*p values versus controls; §p values versus baseline

*Abbreviations*: DME: diabetic macular edema; SMPL: subthreshold micropulse laser; TIMP: Tissue Inhibitor of Metalloproteinase.

**Table 3:** Aqueous humor concentration of proteins significantly increased in patients with diabetic macular edema at baseline versus healthy subjects (controls).

Protein	Controls	DME Baseline	P-value
RANTES	97.8 (62.7)	170.3 (85.0)	**0**.**048**
MIP1α	155.1 (102.7)	285.3 (102.4)	**0**.**021**
FASL	71.6 (116.2)	124.8 (76.0)	**0**.**041**
VEGF	112.8 (121.2)	164.2 (129.8)	**0**.**048**

The results are reported as mean values and standard deviation in brackets of fluorescence intensities signals, measured on glass chip array. P-value = Wilcoxon-Mann-Whitney raw p-value; statistically significant results have been reported in bold characters.

*Abbreviations:* MIP1α = Macrophage Inflammatory Protein α; FasL = fas ligand; RANTES: regulated and normal T cell expressed and secreted; VEGF: vascular endothelial growth factor.

**Table 4:** Changes of proteins’ expression in the aqueous humor of patients with diabetic macular edema at 1, 3, and 12 months after treatment with Sub-threshold Micropulse Laser, compared to baseline.

Protein	Baseline	After 1 month			After 3 months			After 12 months		
	Mean	Mean	Change	P-value°	Mean	Change	P-value°	Mean	Change	P-value°
β2M	4067.5 (3073.7)	3426.0 (2819.3)	−641.5 (746.0)	**0**.**0195**	4951.5 (4198.8)	884.1 (1523.3)		4260.7 (3734.8)	193.2 (1131.8)	
bNGF	347.8 (177.6)	252.8 (157.1)	−95.1 (111.7)	**0**.**0059**	283.4 (173.2)	−64.4 (149.4)		339.3 (221.8)	−8.5 (138.5)	
bFGF	86.9 (46.8)	44.8 (30.6)	−42.1 (48.2)	**0**.**0059**	80.4 (47.2)	−6.6 (73.3)		39.1 (26.2)	−56.3 (58.7)	**0**.**0156**
Eotaxin2	950.7 (891.8)	1039.1 (1298.3)	88.4 (476.2)		165.6 (137.5)	−785.1 (932.8)	**0**.**0019**	757.3 (624.9)	−193.4 (336.2)	
FASL*	124.9 (76.0)	58.7 (14.2)	−66.2 (75.0)	**0**.**0098**	85.5 (40.5)	−39.4 (79.0)		76.6 (39.2)	−48.3 (85.6)	
ICAM1	110.4 (58.1)	46.2 (24.0)	−64.2 (60.4)	**0**.**0039**	96.0 (46.9)	−14.4 (74.5)		65.9 (28.5)	−54.9 (76.6)	
ICAM2	311.7 (363.6)	397.2 (569.6)	85.5 (280.4)		274.7 (400.4)	−37.0 (115.1)		151.3 (182.6)	−160.4 (199.9)	**0**.**0195**
MCP1	35594.7 (27556.6)	37330.9 (24670)	1736.2 (5649.2)		33679.5 (25882.6)	−1915.2 (4245.3)		29158.6 (23858.7)	−6436.1 (7009.9)	**0**.**0195**
MIP1α*	285.4 (102.4)	171.4 (57.3)	−114.0 (72.2)	**0**.**0019**	222.5 (107.1)	−62.9 (79.6)		247.3 (74.3)	−38.1 (105.1)	
MIP3β	622.9 (261.7)	505.9 (321.4)	−117.0 (107.6)	**0**.**0098**	628.0 (339.2)	5.1 (154.2)		496.7 (303.1)	−126.2 (143.8)	**0**.**0156**
RANTES*	170.3 (85.0)	102.9 (25.0)	−96.8 (87.0)	**0**.**0156**	146.0 (125.7)	−24.3 (145.5)		93.4 (40.7)	−76.9 (95.9)	**0**.**0273**
NT4	356.1 223.0	250.1 (138.7)	−106.0 (115.0)	**0**.**0098**	339.4 (259.1)	−16.8 (139.9)		337.7 (181.0)	−18.5 (156.1)	
sTNFRII	952.6 (1149.8)	1276.6 (1774.5)	324.1 (705.8)		886.8 (1032.4)	−65.8 (230.6)		622.9 (584.7)	−329.7 (578.1)	**0**.**0195**
TIMP2	474.2 (782.3)	598.2 (1391.3)	124.0 (649.1)		392.7 (750.1)	−81.5 (182.9)		173.8 (286.7)	−300.4 (510.4)	**0**.**0137**
TIMP3	137.1 (69.7)	82.7 (24.0)	−54.4 (57.7)	**0**.**0234**	113.4 (37.0)	−20.5 (73.7)		80.1 (45.2)	−50.5 (55.6)	**0**.**0391**
TIMP4	125.5 (43.6)	87.3 (52.0)	−36.7 (70.5)		112.2 (66.5)	−22.0 (83.4)		88.2 (41.0)	−37.3 (42.3)	**0**.**0019**
VCAM1	202.6 (154.9)	345.2 (622.0)	124.9 (509.2)		167.1 (186.4)	−35.5 (120.8)		110.3 (109.1)	−92.3 (105.7)	**0**.**0195**
VEGFR2	309.0 (192.6)	353.3 (364.0)	44.4 (205.3)		292.3 (265.5)	−16.7 (131.1)		235.1 (149.6)	−73.9 (87.2)	**0**.**0371**
VEGF *	164.3 (129.8)	190.9 (230.1)	+26.6 (107.1)		127.4 (114.3)	−36.9 (39.6)	**0**.**0106**	134.7 (140.1)	−29.6 (36.6)	**0**.**0456**

The results are reported as mean values and standard deviation in brackets of fluorescence intensities signals, measured on glass chip array. P-values: Wilcoxon-Mann-Whitney test. Significant results have been reported in bold characters.

*Proteins with significantly increased concentration versus healthy subjects.

*Abbreviations*: β2M = β2-microglobulin; bNGF = basic Nerve Growth Factor; bFGF = basic Fibroblast Growth Factor; FAS L = Fas Ligand; ICAM: Intracellular Adhesion Molecule; MCP: Monocyte Chemo-attractant Protein; MIP: Macrophage Inflammatory Protein; RANTES: regulated and normal T cell expressed and secreted; NT = NeuroTrophin; sTNFR = soluble Tumor Necrosis Factor Receptor; TIMP: Tissue Inhibitor of Metalloproteinase converting enzyme; VCAM: Vascular Cell Adhesion Molecule; VEGF: Vascular endothelial growth factor; VEGFR: VEGF Receptor.

## Discussion

Proteomic studies on biological ocular samples, such as AH or vitreous body, have become more common, in recent years, to investigate retinal diseases. The biodynamic of ocular fluids has demonstrated motion of molecules between the two compartments of the eye (vitreous cavity and anterior chamber)^32,33^. Moreover, some Authors have previously demonstrated, in branch retinal vein occlusion and DR, that the aqueous level of VEGF may reflect its vitreous level^34,35^. The feasibility of AH sampling is therefore considered appropriate to study the pathophysiology of many retinal disorders, such as DR and DME, and also to evaluate any changes induced by treatments^19–30^. Considering AH sampling from the anterior chamber safer than vitreous sampling, in this study, we used a proteomic approach on AH to evaluate the effects of SMPL treatment on protein expression. The therapeutic effects of SMPL are completely different from those of continuous wavelength laser photocoagulation (CWL), the latter causing thermal destruction of the retina. While it is the surviving tissue around the retinal scars that probably mediates the efficacy of CWL, SMPL is a tissue-sparing technique. In fact, the thermal elevation does not induce protein coagulation of the retinal tissue^12,13^. It has been hypothesized that SMPL might be able to induce changes of the metabolic activity of the retinal cells, with consequent changes of gene expression and protein secretion^12,18^. Midena *et al*. have already demonstrated that AH biomarkers of Müller cells are influenced by SMPL, suggesting that the metabolic activity of these retinal macroglial cells is improved by this laser treatment^29^. In this study we analyzed 58 proteins, belonging to the inflammatory cascade and produced by several retinal cells, mainly by the MGC. Consistent with a previous paper from our group, comparing patients with and without DR to non-diabetic subjects, we found an increased concentration of some specific inflammatory proteins in diabetic patients with DME^22^. Noteworthy, RANTES, previously reported increased in DR patients^22^, showed an increased concentration in DME group versus non-diabetic controls. RANTES, also known as Chemokine Ligand 5 (CCL5), is a chemotactic cytokine, inducing the recruitment of lymphocytes into inflammation sites. At 1 month and at the end of study its concentration was significantly reduced (p < 0.05 for both measurements, see figure 3). In the present study, also MIP1α and FasL concentrations were significantly increased in DME patients, as already demonstrated in diabetic patients^22,36–38^. MIP1α is an inflammatory cytokine with chemotactic function, usually produced by macrophages in response to bacterial infections and inflammation. As already discussed, MGC are considered the resident macrophages of the CNS and retina. Therefore, one may hypothesize that the major source of MIP1α in the retina is the retinal microglia^24^. In our study MIP1α showed a significant decrease at 1-month post first SMPL treatment (p = 0.002, Fig. 1) and slowly re-increased. Again, FasL, a molecule with pro-apoptotic function, showed a drop down of AH level after SMPL treatment at 1 month from first treatment (p = 0.0097, Fig. 1) and then stabilized. VEGF was also increased in DME eyes versus non-diabetic controls, in accordance with previous findings^19,20^, and showed a significant reduction at 3 and 12 months after treatment (Table 4, Fig. 1). It has been already demonstrated that VEGF is released by several retinal cells and among those, microglia is a considerable source^39^.

The two groups of patients, DME and non-diabetic controls were compared for the major systemic parameters that can influence glial cells activity activity^40^, such as hypertension, dyslipidemia or systemic treatment such as insulin (Table 1). The groups of patients were accurately selected in order to minimize these confounding factors. The homogeneity is confirmed by the absence of statistically significant differences of the main general parameters between the two groups (Table 1), making proteomic results as much uninfluenced as possible by external factors other than SMPL application. Moreover, also ocular conditions, such as posterior vitreous detachment, known to influence the concentrations of local inflammatory molecules, was not present in our patients, neither as a recent event (previous 6 months), nor it verified during the study period^41^. Considering the highly selected study population, the significant reduction of concentrations of the dosed proteins may suggest that SMPL is able to induce a down-regulation of inflammatory retinal processes. Specifically, our proteomic results show a decrease of inflammatory proteins mostly produced by activated retinal microglia. These inflammatory proteins are confirmed increased in diabetic eyes compared to non-diabetic subjects, as previously demonstrated^22–38^. As already mentioned, inflammation has been recently recognized as an important driver of the pathogenesis of DME^4–7^. Our results suggest that SMPL acts by de-activating MGC and by reducing the production of cytokines and chemokines, including VEGF.

We have also found the reduction of Osteopontin, TIMP1 and TIMP2 concentrations in DME eyes (Table 2). Abu-EL Asrar and co-workers^42^ showed an increased concentration of Osteopontin in proliferative diabetic retinopathy, a clinical situation dominated by retinal ischemia. The role of this protein has been more largely investigated in the CNS, specifically in some neurodegenerative disorders, such as Alzheimer’s disease^43^. Osteopontin has both pro-inflammatory and neuro-protective effects, and it is supposed that it modulates the activation and migration of CNS microglia^42,43^. Anyway, the role of osteopontin in DME remains unclear. Moreover TIMP1 and TIMP2, regulators of the metalloproinases’ (MMP) activity, were significantly reduced at baseline in DME eyes, confirming previous results^44^. Metalloproteinases are a group of proteins mainly produced by activated microglia in diabetic retinopathy, which contribute to remodeling the extracellular matrix (ECM); on the contrary, TIMPs (MMPs’ inhibitors) are reduced in DR, leading to imbalanced ECM remodeling^44^. Many other pro-inflammatory cytokines, produced by MGC under stress conditions such as DR and DME (listed in Table 4), showed a significant reduction after the SMPL treatment. Unfortunately, despite their increased mean value in DME eyes at baseline, a statistically significant difference was not fully demonstrated. Moreover, no reliable correlations among retinal thickness changes and proteins’ concentrations were found at each time point, probably because of the low thickness (<400 μm) of the selected DME eyes, and the small sample size. The main limitations of this study, in fact, is the reduced number of eyes. Other limitations of our study, even though minimized by a strict selection of patients, may be represented by possible confounding systemic or local factors that can influence retinal glial activity^40,41,45^, or the degree of lens opacification (cataract) in the non-diabetic control group, compared to DME group, which may theoretically influence AH proteins concentration^46^. Larger studies are needed to confirm our first data.

In conclusion, this study shows, for the first time, the effects of SMPL treatment on protein expression in AH samples of patients affected by DME. The significant decrease of RANTES, MIP1α, FasL and VEGF (pro-inflammatory molecules typically produced by the microglia), suggests that this treatment modality acts by reducing MGC activation. This study contributes to identify possible AH biomarkers of MGC activation and their changes after treatment.

## Data Availability

The datasets used and/or analyzed during the current study are available from the corresponding author on reasonable request.
